# Saffron (*Crocus sativus* L.) and Its By-Products: Healthy Effects in Internal Medicine

**DOI:** 10.3390/nu16142319

**Published:** 2024-07-18

**Authors:** Giulia Marrone, Silvia Urciuoli, Manuela Di Lauro, Kevin Cornali, Giulia Montalto, Claudia Masci, Gianluca Vanni, Manfredi Tesauro, Pamela Vignolini, Annalisa Noce

**Affiliations:** 1 Department of Systems Medicine, University of Rome Tor Vergata, 00133 Rome, Italy; giulia.marrone@uniroma2.it (G.M.); dilauromanuela@gmail.com (M.D.L.); masciclaudia@gmail.com (C.M.); mtesauro@tiscali.it (M.T.); 2 PHYTOLAB Laboratory (Pharmaceutical, Cosmetic, Food Supplement, Technology and Analysis), Department of Statistics, Computer Science, Applications “Giuseppe Parenti” (DiSIA), University of Florence, Via Ugo Schiff 6, Sesto Fiorentino, 50019 Florence, Italy; silvia.urciuoli@unifi.it (S.U.); pamela.vignolini@unifi.it (P.V.); 3 School of Specialization in Nephrology, University of Rome Tor Vergata, 00133 Rome, Italy; giulia.montalto@ptvonline.it; 4 Breast Unit Policlinico Tor Vergata, Department of Surgical Science, Tor Vergata University, Viale Oxford 81, 00133 Rome, Italy; vanni_gianluca@yahoo.it; 5 Nephrology and Dialysis Unit, Policlinico Tor Vergata, 00133 Rome, Italy

**Keywords:** circular economy, *Crocus sativus* L., by-products, natural bioactive compounds, metabolic syndrome, chronic kidney diseases, depression

## Abstract

*Crocus sativus* L., commonly known as saffron, is a precious spice coming from Asia, in particular from Iran, the country leader in its production. The spice is derived exclusively from dried stigmas and it is the most expensive one in the world. The areas of application of saffron are multiple, in fact ranging across the food, drinks, pharmaceuticals and cosmetics sectors. As is the case with other phytochemicals, not only the final product but also saffron by-products are considered a valuable source of bioactive natural compounds. In fact, its healthy effects, especially as antioxidants and anti-inflammatories (via reducing pro-inflammatory cytokines), are well-recognized in internal medicine. In particular, its healthy effects are related to counteracting degenerative maculopathy, depression and anxiety, neurodegenerative diseases, metabolic syndrome, cancer and chronic kidney disease, by promoting glucose metabolism. In this review, we summarize the most important papers in which saffron has turned out to be a valuable ally in the prevention and treatment of these pathologies. Moreover, we would like to promote the use of saffron by-products as part of a bio-circular economy system, aimed at reducing wastes, at maximizing the use of resources and at promoting environmental and economic sustainability.

## 1. Introduction

**Figure 1:**
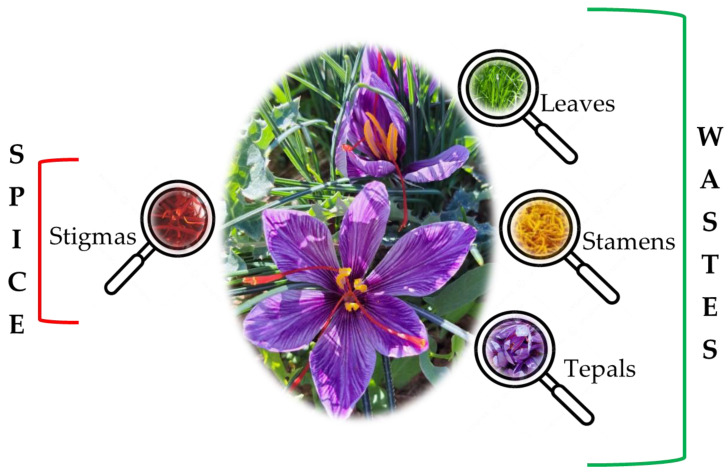
Parts of *C. sativus* L.

Saffron (*Crocus sativus* L.) is one of the most precious spices in the world. It is mainly used in cooking but since ancient times, it has been used in traditional medicine for its healthy properties such as antimicrobial, antispasmodic, aphrodisiac, antibacterial, antifungal, anti-inflammatory and anticancer [1]. Over the years, several studies have tested the effects of saffron against diabetes, macular degeneration, cognitive impairment, glaucoma, sexual dysfunction and premenstrual syndrome [2]. *C. sativus* L. is a plant composed of a bulb, leaves and flower which is composed of tepals, stamens and stigmas. The spice is obtained only from dried stigmas. Saffron is one of the most expensive spices in the world [3]. The main sectors of use are food, drinks, pharmaceuticals and cosmetics [4]. The annual world production of saffron is 178 tons; the leading world producer and exporter is Iran with 80%, while the remaining 20% is represented by India, Morocco, Greece, Spain, Italy, Turkey, France, Pakistan, China, Japan and Australia [1]. The global market value is USD 602.2 million with 7% growth prospects between 2024 and 2030 [5]. To obtain 1 kg of dried saffron, approximately 500,000 stigmas are needed, and therefore, 53 kg of tepals, 1500 kg of leaves and 63 kg of flowers are also obtained [2,6]. The high cost of this spice is due to the manual harvesting of the flowers and the separation of the stigmas from the yellow stamens and violet tepals. The high price and manual labor required to obtain even a small amount of saffron make this industry vulnerable to fraud. This danger resulted in the creation of the ISO3632 standard updated in 2011 to certify the authenticity and quality of saffron. Stigmas and powder must meet laboratory standards including for color, flavor and aroma. These three parameters are directly linked to the presence of three molecules such as crocins, picrocins and safranal. The quality of saffron is also linked to the quantity of crocins and is classified into four classes (class I crocins > 200 and class IV crocins < 120) [7]. The price of saffron depends on these evaluations [8]. To produce saffron (red stigmas), many by-products and wastes are also obtained. Figure 1 shows the unused parts of *C. sativus* L.: leaves, stamens and tepals. These by-products represent a source of nutrients such as proteins, lipids, carbohydrates, calcium, potassium and sodium [9,10]. In this review, we will focus on the natural bioactive compounds of stigmas, tepals, stamens and leaves of *C. sativus* L. in internal medicine. Saffron wastes are rich in phenolic compounds which have positive effects on human health [11]. The valorization of by-products allows the application of a circular model to the saffron supply chain, in order to obtain functional secondary raw materials to be used in the food, pharmaceutical, cosmetic, agronomic and feed sectors. In recent years, several papers have been published regarding the valorization and by-products and wastes from saffron (spice) production, in particular relating to the tepals which account for 80% [2,12,13]. The use of saffron by-products is part of a bio-circular economy system, which aims to reduce wastes, maximize the use of resources and promote environmental and economic sustainability. Supporting agriculture and the saffron industry, at a local level, can help to create a more resilient and self-sustaining circular economy model.

## 2. Search Methods

Four online databases (PubMed, Scopus, Science Direct and Cochrane Library) were used for the literature search. The search was conducted according to the following relevant terms: “saffron” and “*Crocus sativus* L.” in combination with “wastes” AND “by-products” AND “compounds” AND “composition” AND “characterization” AND “internal medicine” AND “degenerative maculopathy” AND “metabolic syndrome” AND “depression” AND “anxiety” AND “cancer” AND “neurodegenerative disease” AND “chronic kidney disease”. The full search was manually retrieved. The studies included in this paper are limited to review and original articles, written in the English language. The collected articles were published between 1990 and 2024.

## 3. Saffron Natural Bioactive Compounds

**Figure 2:**
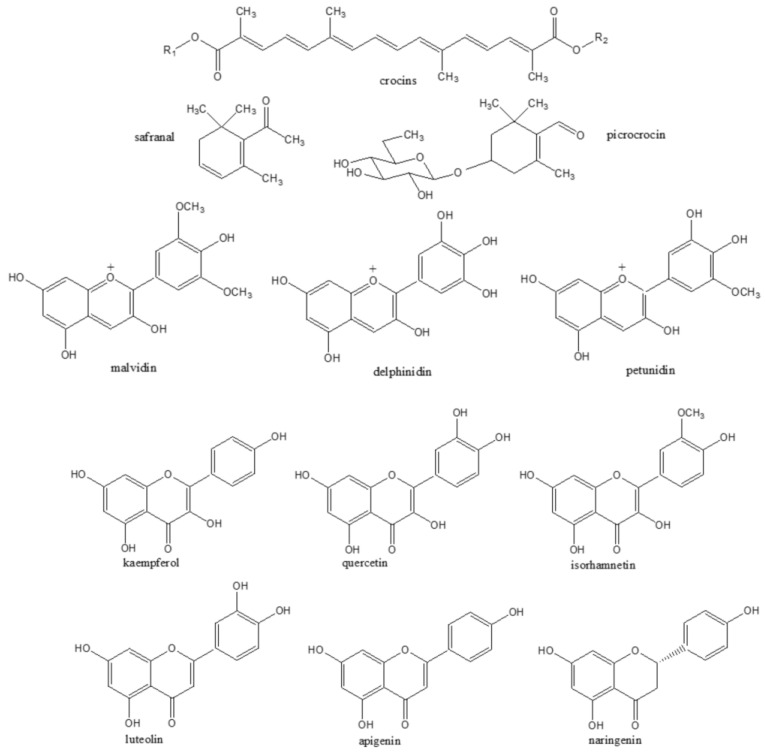

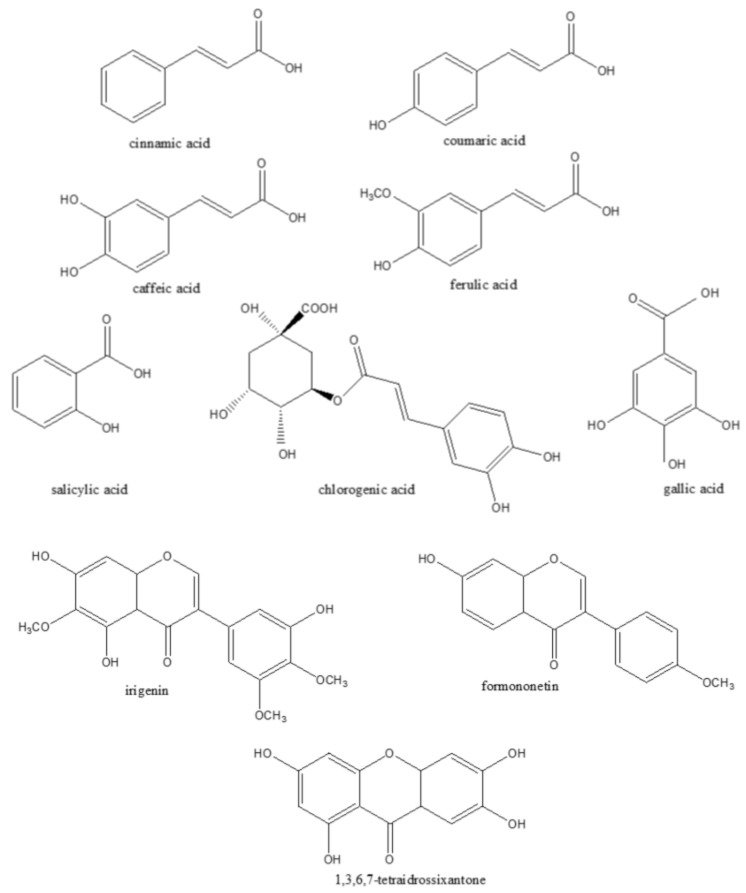
Main saffron bioactive compounds.

Saffron production generates a large amount of waste products. To produce 1 kg of dried saffron stigmas, between 110,000 and 170,000 flowers are needed, which involves a high quantity of waste which includes tepals, stamens and leaves [14,15,16]. The chemical characterization of *C. sativus* L. highlights not only the dry stigmas, but also the floral bio-residues which represent about 93% of the weight of the entire flower [14]. The different parts of *C. sativus* L., such as the stigmas, tepals, stamen and leaves contain different phytochemicals such as carotenoids, flavonoids, anthocyanins and phenolic acids [17] (Figure 2). 

### 3.1. Saffron Stigmas 

The stigmas are characterized by the presence of water, protein, minerals, sugars (reducing sugars, pentosans, gums, pectin and dextrins), fiber, vitamins (such as riboflavin and thiamine) and secondary metabolites including terpenes, flavonoids and carotenoids [18,19,20,21]. The content of all the compounds depends on the country in which *C. sativus* L. is grown, the climate and the drying and storage conditions [22].

Many analytical studies have been conducted to characterize the potential biological active compounds of saffron and the main ones are crocins, safranal and picrocrocin [23,24]. 

Crocins are responsible for the characteristic color of saffron. Crocins are hydrophilic carotenoids, which include mono- or di-glycosyl polyene esters of crocetin [23]. Carmona et al. [25] identified fifteen crocin esters: trans or cis crocetin esters with a number of glucose moieties from one to five. Even lycopene, α- and β-carotene and zeaxanthin, which represent lipophilic carotenoids, were found in trace amounts [20].

Picrocrocin (4-β-D-glucopyranosyloxy-2,6,6-trimethyl-1-cyclohexen-1 carboxaldehyde) is responsible for the unique taste of the saffron spice, and it is a degradation product of zeaxanthin [26]. During the enzymatic process, picrocrocin loses glucose and is transformed into safranal (2,6,6 trimethyl-l,3-cyclohexadien-l-carboxyaldehyde) which is responsible for saffron’s smell [27]; it is not present in fresh stigmas and it occurs after stigmas’ drying and storage [25]. Sixty volatile compounds were detected in saffron stigmas, and safranal is the most abundant [28]. 

Even flavonoids were identified in saffron stigmas. In particular, Carmona et al. [25] identified kaempferol-3-sophoroside, kaempferol-3-sophoroside-7-glucoside and kaempferol-3,7,4′-triglucoside, as confirmed by Vignolini et al. [16], and other compounds, such as kaempferol tetrahexoside and kaempferol-3-dihexoside were detected. However, Menighini et al. [29] did not highlight the presence of kaempferol derivatives, but they reported quercetin, rutin and naringin.

### 3.2. Saffron By-Products

Saffron flowers, together with floral bioresidues from saffron spice production, are sources of interesting bioactive compounds. In particular, saffron tepals contain different classes of flavonoids including flavonols and anthocyanins. As regards the flavonol fraction, the main compounds are kaempferol, quercetin and isorhamnetin derivatives, in particular, mono-, di- and triglycoside, with kaempferol 3-O-sophoroside being the most abundant [14,30]. A class of compounds typical of saffron tepals is the anthocyanins, which are responsible for their purple color. The principal anthocyanins detected are delphinidin, petunidin and malvidin derivatives, and in particular, mono and di glucosides, pelphinidin 3,7-O-diglucoside, delphinidin 3-O-glucoside, petunidin 3,7-O-diglucoside, petunidin 3-O-glucoside and malvidin O-glucoside were detected by Goupy et al. [30] and Moshfegh et al. [31], with delphinidin 3,7-O-diglucoside being the most abundant with 3.93 mg/g. Other authors have instead evidenced the presence of delphinidin 3,5-O-diglucoside, petunidin 3,5-O-diglucoside and malvidin 3,5-O-diglucoside [14,32,33]. Lutein esters with lauric, myristic, palmitic and stearic acids were even found in *C. sativus* L. flowers [30]. Some authors have also highlighted traces of crocins [16,34].

*C. sativus* L. stamens even showed the presence of flavonols like kaempferol, quercetin and isorhamnetin glycosides, with kaempferol 3-O-sophorodside being the most abundant compound [16,34]. Furthermore, small amounts of crocins were highlighted [16].

Although the leaves represent a by-product in the production of the saffron spice, there are few papers regarding the characterization of this matrix, and the few data from the literature on the chemical composition of *C. sativus* L. leaves are very contradictory. Over 40 samples of *C. sativus* L. leaves from nine different countries on different continents (Asia, Africa, Oceania, Europe) were analyzed using HPLC/DAD/MS analysis, in particular, Smolskaite et al. [35] evidenced for the first time the presence of kaempferol-8-C-gluco-6-O-glycoside, kaempferol-8-C-gluco-6,3-O-diglycoside, as well as quercetin-8-C-glycoside, luteolin-8.3-c-diglycoside, quercetin-3-O-maltotrioside, luteolin-8-C-glycoside, kaempferol-3-O-sophoroside and apigenin-8-C-glycoside. Even naringin was found by Baba et al. [36]. The HPLC/DAD/MS analysis of *C. sativus* L. leaves from Morocco evidenced the glycosylated derivatives of luteolin (luteolin-C-[(O-caffeoyl-hexosyl)-O-hexoside) and glycosylated kaempferol (kaempferol 3,7-di-O-glucoside) as the major identified polyphenols in the leaves [37]. Tajika et al. [38] identified gallic acid, cinnamic acid, caffeic acid, coumaric acid, salicylic acid, ferulic acid, quercetin and kaempferol, while Mykhailenko et al. [39] detected for the first time in saffron leaves five phenolic compounds: chlorogenic acid, mangiferin, ononin, trans-cinnamic acid and irigenin.

## 4. Beneficial Effects of Saffron in Internal Medicine

**Figure 3:**
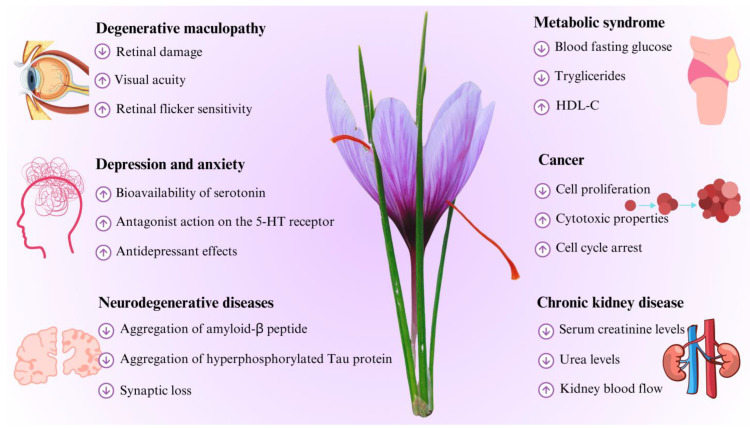
Potential beneficial effects of *C. sativus* L. in internal medicine. Abbreviations: ↑, increase or improvement; ↓, decrease; 5-HT, serotonin; HDL-C, high-density lipoprotein cholesterol.

All parts of saffron, both the stigmas and saffron by-products (such as leaves, tepals and stamens), appear to have numerous beneficial effects in internal medicine (Figure 3). In particular, we examined the beneficial effects in degenerative maculopathy (DM), depression and anxiety, neurodegenerative disease, metabolic syndrome (MetS), cancer and chronic kidney diseases (CKDs) (Figure 3).

### 4.1. Degenerative Maculopathy

DM is a neurodegenerative disease affecting the retina, and its development is caused by both genetic and environmental factors. However, a crucial role seems to be played by oxidative stress (OS) [40] and chronic inflammation. In particular, OS is involved in many age-related chronic neurodegenerative diseases [41], whose progression is accelerated by the cellular damage induced by the reactive oxygen species (ROS). 

Compared with other tissues, ROSs are the most produced species in the retina because of the oxidable fatty acids, present in photoreceptors, but also because of the photosensitizers occurring in the retinal pigment epithelium (RPE).

DM is characterized by hyper or hypopigmentation of the RPE that leads to an apoptotic process, which involves photoreceptors. The main consequence of this process is visual impairment. For this reason, several studies, including the Age-Related Eye Disease Study (AREDS) [42], have evaluated the possible beneficial effects induced by natural supplements on DM. Natural compounds (such as zinc, vitamin C and β-carotene) have been shown to reduce the DM progression in its early stages, protecting the retina and its epithelium from damage [43]. Other compounds, like lutein and zeaxanthin carotenoids, both antioxidants [44], are retina constituents, and they seem to have a positive effect on the DM progression, by protecting the macula structure from damage.

Among all these bioactive compounds, those that constitute saffron could also play a key role in slowing down the progression of DM.

Crocetin, a main constituent of *C. sativus* L., has been analyzed in several experimental studies. In particular, Ohno et al. [45] highlighted its beneficial effect in mouse models on N-methyl-D-aspartate receptor (NMDA)-induced damage. Crocetin oral supplementation ameliorates retinal damage and counteracts cells apoptosis by inhibiting the caspases’ expression.

Another randomized trial, which included 29 patients in the early DM stage, highlighted the beneficial effect of saffron in macular tissue; the saffron intake (20 mg per day) in fact, improves the visual acuity and slows down the DM clinical progression [46].

Moreover, also carotenoids, powerful antioxidants, exert a positive effect on retinal flicker sensitivity [43]. 

Therefore, saffron-based food supplements could represent a valid adjuvant strategy to slow down the progression of DM.

### 4.2. Depression and Anxiety 

Depression and anxiety are the most common psychiatric pathologies in the world. The main antidepressants act by increasing the bioavailability of serotonin, the main neurotransmitter with a physiological role in humans [47]. In spite of that, its action is not specific and these drugs may not have an immediate effect, but rather have a late effect with a large number of side effects (such as an excessive sensation of agitation, tremors, states of anxiety or gastrointestinal disorders) [48]. In this context, new adjuvant treatments based on natural compounds, free of side effects, can have a fundamental role to counteract depression and anxiety. In this context, saffron and its derivatives, free of side effects and with potential beneficial effects in modulating mood, seem to be very interesting [49,50]. In particular, it has been demonstrated that saffron is able to exert strong anti-depressant effects, potentially due to its antioxidant, anti-inflammatory and serotonergic action [51].

In patients suffering from depression, an increase in OS is observed, which in turn is related to the alteration of the immune system and to the neurotransmitters’ balance alteration, contributing to neurodegeneration. A recent meta-analysis confirmed how the daily use of saffron, in different forms, is able to reduce OS in patients with depression, highlighting a concomitant increase in the activity of antioxidant enzymes (such as superoxide dismutase-SOD, catalase-CAT and glutathione peroxidase), activities that are usually altered in patients with depression [51].

Depression is known to be associated with neuroinflammation and with increased levels of C-reactive protein, interleukin-6 (IL-6) and tumor necrosis factor (TNF)-α. Safranal, crocin and crocetin act synergistically to exert anti-inflammatory effects by reducing pro-inflammatory cytokines [52].

During a depressive disorder, there is also an alteration in the serotoninergic system with the reduction in tryptophan levels, the precursor of serotonin, responsible for depressive symptoms. Saffron appears to be able to increase the bioavailability of serotonin, although the exact mechanism has not yet been discovered [53]. It is hypothesized that crocin exerts an antagonistic action on the site of the 5-HT 2c receptor, the serotonin receptor found on the membrane of neurons [54]. 

The anti-depressant effects have been studied not only for saffron but also for the other parts of *C. sativus* L. In particular, a study conducted by Hosseinzadeh et al. highlighted the antidepressant effects of kaempferol, a constituent of the *C. sativus* L. petal, in murine models [55].

The antidepressant effects of saffron have been evaluated through numerous case/control clinical trials in humans. In particular, in a double-blind study conducted by Mazidi et al. [56], the adult patients with anxiety and depression enrolled were randomized into two groups, wherein one consumed 50 mg of saffron stigmas for 12 weeks and the other consumed a placebo, for 12 weeks. At the end of the study, an improvement, compared to the control group, was observed in the Beck Depression Inventory (BDI) and Beck Anxiety Inventory (BAI) questionnaires administered, confirming the anti-depressant effects of saffron.

### 4.3. Neurodegenerative Diseases

The different components of *C. sativus* L. appear to be able to counteract the main neurodegenerative diseases that affect the geriatric population, namely Alzheimer’s and Parkinson’s diseases [57]. In this regard, as saffron is rich in numerous bioactive molecules that act synergistically (mainly crocin, crocetin and safranal), it seems to be capable of counteracting neuroinflammation, modulating the metabolic pathways of autophagy and apoptosis and reducing the OS, all phenomena present in neurodegenerative diseases [58].

In fact, several clinical trials, conducted on murine models with induced alterations that mimic some of the neurodegenerative disease symptoms, suggest that saffron inhibits the production of pro-inflammatory cytokines, induces antioxidant effects with the reduction in ROSs, exerts anti-apoptotic actions in the brain cells and improves the mitochondrial function with a consequent neuroprotection [59,60,61].

Alzheimer’s disease is characterized by a progressive loss of memory and learning functions, and it is the most common cause of dementia, especially in the elderly population. It is characterized by structural brain alterations, such as the formation of amyloid plaques, which impair brain capacity [62]. Saffron appears to exert neuroprotective effects against the cognitive deterioration caused by Alzheimer’s disease. In particular, these effects, demonstrated in in vitro studies, are employed by crocin and crocetin [48]. Both molecules inhibit the aggregation of the amyloid-β (Aβ) peptide and slow down the synaptic loss [63]. Furthermore, crocin and crocetin, also obtained from the circular economy models, act by attenuating the OS, the neuroinflammation and the apoptosis of neuronal cells in mouse models with Alzheimer’s disease [64].

Furthermore, in vivo studies have demonstrated how trans-crocetin, i.e., a biologically active metabolite of crocin, is able to prevent the formation of senile plaques and neurofibrillary tangles (NFTs) and intracellular aggregations of hyperphosphorylated tau protein, namely the protein commonly known as the primary biomarker causing Alzheimer’s disease, through the suppression of acetylcholinesterase (AChE) activity [65,66].

However, few studies have been conducted on humans to evaluate the neuro-protective effect of saffron and its derivatives in vivo. An interesting and recent meta-analysis had as its primary aim to evaluate the cognitive function and the quality of life of Alzheimer’s disease patients, who consumed the saffron in any form (namely powder, extract or oil). This meta-analysis confirmed the neuroprotective effects of saffron, thanks to the significant improvement in cognitive function as assessed by the Alzheimer’s Disease Assessment Scale-cognitive subscale (ADAS-cog) and the Clinical Dementia Rating Scale-Sums of Boxes (CDR-SB), compared to the control group [67].

Thus, this evidence suggests how the regular intake of all the parts of saffron can contribute to the attenuation and to the control of the most common neurodegenerative diseases of our era.

### 4.4. Metabolic Syndrome 

MetS is a chronic pathology that requires significant attention due to its complicated nature. MetS is defined by the International Diabetes Federation (IDF) criteria, elaborated in 2005 [68], for the presence of central obesity (defined as a waist circumference ≥ 94 cm in the males and ≥80 cm in the females of the Caucasian population), plus almost two of the following criteria: increased triglycerides levels (≥150 mg/DL) or specific treatment of this lipid abnormality; decreased high-density lipoprotein cholesterol (HDL-C) levels (≤40 mg/dL in men and ≤50 mg/dL in females or the specific treatment of this lipid abnormality; increased blood pressure levels (≥130/85 mmHg) or in treatment with antihypertensive drugs and impaired fasting glucose (≥100 mg/dL) or a previous diagnosis of type 2 diabetes mellitus [69]. The development of this pathological condition may depend on both genetic factors and lifestyle, such as unhealthy eating habits and physical inactivity [70,71].

Several studies have highlighted the beneficial effects of *C. sativus* L. and its bioactive compounds on the metabolic profile, mainly thanks to their antioxidant capacity [72,73,74]. Indeed, the antioxidant potential of all the parts of saffron (tepals, stamens, styles and stigmas) has been highlighted through numerous in vitro assays (including lipid peroxidation, deoxyribose assay, Rancimat test, and Trolox equivalent antioxidant capacity), and this could have positive repercussions for human health [73]. In support of this evidence, a study conducted by Mohammadi et al. [75] demonstrated that saffron and its components are able to increase the total antioxidant capacity in diabetic rats. In particular, the antioxidant action is exerted by the reduction in serum malonyldialdehyde.

Other in vivo studies conducted in animal models demonstrated saffron antidiabetic proprieties, thanks to the positive impact of its bioactive compounds on glycemia. These effects include the improvement in fasting blood glucose, the reduction in serum insulin and HbA1c levels and the decreased production of advanced glycation end products (AGEs) [76,77,78,79].

Some researchers have recently investigated the role of *C. sativus* L. as an alternative and adjuvant treatment to decrease the high costs incurred in diabetes mellitus care [80]. In particular, in a randomized placebo-controlled trial, conducted on MetS patients over a period of 12 weeks [81], the authors investigated the effect of saffron supplements on the oxidant–antioxidant balance. In detail, 75 MetS patients were randomly divided into two groups: the first group consumed 100 mg/kg saffron for 12 weeks, while the second group represented the control group (standard care). At the end of the study, a significant improvement in the serum oxidant–antioxidant balance was detected. The authors hypothesized that saffron supplements may modulate the redox balance in MetS patients.

Another study, conducted on elderly hypertensive patients, examined the combined effects of saffron and resistance training on the inflammation biomarkers and on the cardiovascular risk factors [82]. The study lasted 12 weeks and was conducted on 48 hypertensive male patients, aged between 60 and 70 years, who were randomly assigned to a control group (C) or to one of the three intervention groups, saffron consumption (S), resistance training (R) or resistance training + saffron consumption (RS). At the baseline and after 12 weeks, the inflammatory markers and lipid profiles were measured. The authors highlighted that the RS group showed significant reductions in leptin, resistin, monocyte chemoattractant protein-1 (MCP-1) and IL-6 compared with the C, S and R groups. The RS, S and R groups showed significant reductions in the total cholesterol (TC) and greater increases in HDL-cholesterol, compared with the C group, but without differences between the RS, S and R groups. This study pointed out how the combination of saffron and resistance training can reduce the inflammation and cardiovascular disease risk factors in older hypertensive men.

Another criterion to diagnose MetS is dyslipidemia. In a double-blind placebo controlled clinical trial [83], 40 patients (with at least two of the following criteria: low HDL-C levels, high low-density lipoprotein cholesterol (LDL-C), high triglyceride (TG) levels or high TC levels) were randomized into two groups: 21 patients consumed saffron petal supplements for 4 weeks at a dose of 30 mg/day, while the other 21 patients consumed a placebo. At the end of the study, the parameters such as serum lipid factors, alanine transaminase, aspartate transaminase, alkaline phosphatase, urea, creatinine and FBS were analyzed and compared with their values before the intervention.

The results showed a marked decrease in the serum lipid levels (like TG, TC and LDL cholesterol) in the intervention group compared to the placebo group. The results highlighted that saffron petal supplementation reduced significantly the lipid profile in patients with dyslipidemia. Thanks to its properties, saffron could be used as an adjuvant treatment for the clinical management of MetS patients.

### 4.5. Cancer 

Saffron has been described by several studies as a phytochemical useful in the prevention and treatment of cancer. In fact, the stigmas and the saffron by-products, namely petals and leaves, have remarkable anticancer properties, which has been tested both in vivo and in vitro [84]. 

The anticarcinogenic effects of saffron stigmas have been highlighted by numerous studies on several types of cancer [85]. In the in vitro study conducted by D’Alessandro et al. [86], the incubation of prostate cancer (PC) cell lines, such as the PC3 cells, at different concentrations of a saffron stigma extract or crocin extract (CE), was able to reduce the cell proliferation in a time- and concentration-dependent manner. Moreover, the authors highlighted how the CE was capable of inhibiting the cell proliferation, through an increased expression of B-cell lymphoma protein-2 (Bcl-2) (proapoptotic protein) and through an upregulation of Bcl-2-associated X protein (BAX) (antiapoptotic protein), of arresting the cell cycle in the G0/G1-phase and, finally, of inducing apoptosis, through an increase in caspase-3 levels [86]. In a subsequent in vitro study conducted by Ahmadnia et al. [87], the anticancer effect of a saffron stigma aqueous extract (SSAE) on PC3 cells and on mouse fibroblast cells (non-cancerous control cells), was found to be made possible thanks to its antiproliferative properties. Gezici [88] assessed in vitro the cytotoxic and the antiproliferative properties of an SSAE, of a saffron stigma ethanol extract (SSEE) and of crocetin and their effects on the lactate dehydrogenase (LDH) enzyme activity in human non-small lung cancer (A549) cells, in breast adenocarcinoma (MCF-7) cells and in cervical cancer (HeLa) cells, compared to the nonmalignant human vein endothelial (HUVEC) cells.

The author pointed out how the crocetin exerted the higher cytotoxic and antiproliferative activity in the HeLa cells and the greater LDH enzyme release from the damaged HeLa cell membranes, compared to both extracts. The possible in vitro anticancer effects of an SSEE were also evaluated by Samarghandian et al. [89] in A549 cells in comparison with non-malignant (L929) cells. The authors demonstrated a decreased A549 cell viability and a change in their morphology, so as to affirm the possible antiproliferative and cytotoxic effects of the SSEE. Similar effects in A549 cells were also detected by Chen et al. [90], demonstrating how the CE is able to induce morphological changes in the cells, to inhibit the cell proliferation, to provoke the cell cycle arrest in the G0/G1-phase, to increase the p53 mRNA levels, to decrease the Bcl-2 mRNA levels and to exert synergetic or additive antitumor effects on the cell proliferation, when the crocin was combined with cisplatin or with pemetrexed. The in vitro effects derived from the combined chemotherapy treatment have been better studied by Lozon et al. [91] in A549 cells and in colon cancer (HCT116) cells. The authors demonstrated how the safranal can synergistically or additively enhance the cell growth inhibitory effects attributable to topotecan (TPT), especially when administered before TPT. Moreover, the combined treatment increased in both cell lines the amount of DNA double-strand breaks and reduced the expression of the tyrosyl-DNA phosphodiesterase 1 enzyme (responsible for repairing DNA lesions), compared to the cells treated with TPT alone. In conclusion, the authors observed how the DNA damage to the A549 and to HCT116 cells did not permit them to enter the G2/M-phase, so as to be removed by apoptosis. Luo et al. [92] evaluated adenocarcinoma gastric (AGS) cell lines the in vitro effects induced by incubating cells with crocin. The authors pointed out how the crocin was able to inhibit the AGS cells’ proliferation through the downregulation of the tropomyosin alpha-4 chain (TPM4) gene expression. 

In addition to the potential anticancer properties possessed by the saffron stigma, few studies have evaluated the biological activity of the secondary metabolites contained in the saffron waste products. Mykhailenko et al. [93] tested the cytotoxic activity of the water and ethanolic extract from saffron leaves against a human melanoma-derived (IGR39) cell line and triple-negative breast cancer (MDA-MB-231) cell line. Kaempferol and quercetin were the most cytotoxically active compounds against both cell lines, mangiferin and rutin were the two phenolic compounds with the highest antioxidant activity, while chlorogenic acid and ferulic acid showed the highest affinity for several breast cancer proteins, comparable to that of hydroxytamoxifen. 

Concerning saffron petals, Tu et al. [94] assessed in vivo the anti-tumor effects of saffron petals’ polysaccharides in a saline solution (at the dose of 2.5, 5.0 and 10 mg/kg) for 12 days in the reconstructed tumor microenvironment of the S180 sarcoma-bearing mice. In the sarcoma tissues, the authors pointed out: (i) an induction of tumor cell necrosis, apoptosis and vessel disruption; (ii) a reduction in the levels of inflammatory factors (transforming growth factor β-TFG-β, interferon γ-IFN-γ, IL-10, TNF-α); (iii) a recruitment of anti-tumor immune cells (CD8+ T cells and natural killer cells); (iv) a reduction in the pro-tumor immune cells (regulatory T cells-Tregs, total myeloid-derived suppressor cells (MDSCs) and tumor-associated macrophages (TAMs)); and (v) a reprogramming of the TAMs from a tumorigenic M2 into an antitumorigenic M1 phenotype [94]. 

Finally, in the study conducted by Sharma et al. [95], a saffron petal extract (SPE), prepared from 50 g of dried petal powder and extracted using 100 mL of n-butanol, was tested in vitro against a liver cancer (Huh-7) cell line. It was found that the SPE contained a high number of phytochemicals (such as tannins, saponins, alkaloids, phenols, etc.) and of phenolic and flavonoid compounds, indicating its radical scavenging activity, assessed through the 1,1-diphenyl-2-picrylhydrazyl (DPPH) assay and ferric reducing antioxidant power (FRAP) assay, and its potential cytotoxic activity against a Huh-7 cell line. 

Moreover, Vago et al. [96] in 2023, tested the activity of saffron extracts on kidney (RT4) and bladder (RT112) cancer cell lines, along with crocin and safranal standards. They showed a significant reduction in the cell viability with saffron extracts, while the standards alone did not exert any cytotoxicity. The mixed solution between the saffron extract and standard showed a milder inhibition, indicating a synergistic effect of the phytocomplex. At the same time, a saffron flower extract was also tested on kidney and bladder cancer cells which showed a reduction in the cell viability results.

In conclusion, saffron and its by-products, namely leaves and petals, can be used as an adjuvant therapy to the pharmacological ones. However, further in vivo studies are required to understand both the biological effects on humans and the right posology of saffron in order to exert its potential anticancer effects. It is important to underline how the saffron extract only induced cell death in the cancer cell lines, thus suggesting its anticancer activity with no cytotoxic effect on normal and on nonmalignant cells. 

### 4.6. Chronic Kidney Disease 

CKD is a chronic clinical condition, which has been showing in recent years an increase in its worldwide prevalence [97].

In CKD patients, a key role in the kidney dysfunction process is played by chronic inflammation [98,99]. However, among the factors leading to the development of a low-grade chronic systemic inflammatory state, there are OS, uremic toxins’ accumulation and hypoxia [100]. OS causes cellular damage that results in tissue dysfunction and organ failure. In CDK, ROS accumulation promotes structural and function damage that worsens the clinical picture [101,102]. Therefore, the kidney loses its ability to filtrate the toxic compounds that accumulate. For this reason, the early identification of these mechanisms can prevent the progression of kidney damage. 

In this regard, there have been numerous studies that affirm how natural food supplements exert positive effects on many of the chronic degenerative non transmissible diseases. In fact, saffron and its bioactive compounds exert numerous beneficial effects in many pathologies, including CKD [103]. Thanks to the presence of some bioactive compounds, such as crocetin and crocin, saffron induces its antioxidant, anti-inflammatory actions, promoting kidney health [104]. 

In particular, a study on an animal model conducted by Samarghandian et al. [105] showed that crocetins are able to decrease the OS through the increase in serum nitric oxide (NO) and malondialdehyde, and through the increase in glutathione S-transferase (GST) activity. Moreover, also safranal has shown the same effects [106].

Other studies have demonstrated that the carotenoids and flavonoids contained in saffron, can be useful to counteract free radicals’ production [107]. In detail, an interesting study conducted by Karimi et al. [108] hypostatized that the saffron antioxidant action can be attributed to the synergistic effect between phenolic compounds and flavonoids.

Several studies suggest that saffron intake leads to an improvement in serum creatinine levels [106]. This positive effect can be ascribed to the increase in the renal blood flow. In fact, crocin seems to exert a renal vasodilator effect, that induces a decrease in serum creatinine through the increase in oxygen delivery at the renal level [109]. Moreover, saffron can induce a diuretic action on distal convoluted tubules, through the inhibition of sodium and chloride symport [110].

In conclusion, saffron and its compounds could represent a valid strategy in counteracting the CKD onset and progression, as demonstrated by the numerous studies previously mentioned.

## 5. Potential Toxic Effects of Saffron on Health

Although saffron has been shown to be a safe plant for adjuvant use also in association with a pharmacological therapy, as described in the previous paragraphs, there are also some data in the literature on its potential side effects [111]. Such effects include nausea, poor appetite, headache, dry mouth, insomnia and dizziness [20]. In particular, a review article conducted by Schmidt et al. [112] demonstrated that saffron induced toxic effects if consumed in doses higher than 10 g/day, while other studies demonstrated that a consumption of up to 4 g/day for several days induced no toxicity. Another study conducted by Modaghegh et al. [113] tested the potential effects induced by 400 mg/day tablets of saffron for seven days. The authors found a slight reduction in hemoglobin, hematocrit, red blood cells and platelets and an increase in serum sodium, urea and creatinine. Nevertheless, those changes were into the normal range of values, without clinical implications.

Regarding its potential allergic effect, limited data have been reported [114].

Therefore, we can conclude that saffron can be considered safe at the dosage normally used as a spice, as the quantity actually consumed is significantly lower than that which causes side effects.

## 6. Conclusions

The results collected in this review allow us to state that the recovery of waste from the saffron supply chain can represent a sustainable resource of low-cost bioactive molecules in response to the European community’s challenge for a sustainable future. The circular recovery model used for saffron tepals, stamens and leaves can also be applied to other food chains. The value of agri-food wastes and by-products could assure food security, maintain sustainability, efficiently reduce environmental pollution and provide an opportunity to earn additional income for industries [115]. Moreover, this review underlines the potential beneficial effects of saffron and its waste in several internal medicine diseases. Therefore, saffron represents an innovative potential adjuvant therapy in combination with the traditional treatment in the management of the main chronic degenerative non-communicable diseases.
